# Involvement of estrogen in phosphorus-induced nephrocalcinosis through fibroblast growth factor 23

**DOI:** 10.1038/s41598-020-61858-7

**Published:** 2020-03-17

**Authors:** Satoshi Takasugi, Miho Shioyama, Masami Kitade, Masashi Nagata, Taketo Yamaji

**Affiliations:** Division of Research and Development, Meiji Co., Ltd., Tokyo, 192-0919 Japan

**Keywords:** Endocrinology, Phosphorus metabolism disorders

## Abstract

Excessive phosphorus intake adversely affects bone and mineral metabolism. Estrogen is one of the factors affecting fibroblast growth factor 23 (FGF23), a phosphorus-regulating hormone. However, the interaction between excess phosphorus and estrogen status has not been fully elucidated. This study investigated the involvement of estrogen in the effects of high phosphorus intake on bone metabolism and ectopic calcification in ovariectomized (OVX) rats. The interaction between high phosphorus diet and OVX was not observed in bone mineral density and aortic calcium. In contrast, high phosphorus intake markedly increased renal calcium concentration in sham rats, whereas the effect was attenuated in OVX rats, which was reversed by a selective estrogen-receptor modulator treatment. A strong positive correlation between renal calcium and serum FGF23 was observed. In addition, fibroblast growth factor receptor 1 (FGFR1: a predominant receptor of FGF23) inhibitor treatment partially decreased renal calcium concentrations in rats with high phosphorus intake. In conclusion, the effect of high phosphorus intake on bone metabolism and aortic calcification did not depend on the estrogen status; in contrast, high phosphorus intake synergistically induced nephrocalcinosis in the presence of estrogenic action on the bone. Furthermore, FGF23 was involved in the nephrocalcinosis induced by high phosphorus intake partially through FGFR1 signaling.

## Introduction

Phosphorus is one of the nutrients that are more likely to be consumed excessively. According to the NHANES 2005–2006, the mean daily phosphorus intake exceeds the recommended dietary allowance for most age groups^1^. Although the phosphorus content of Western diet is increasing because of the increasing use of phosphorus-containing food additives and increasing consumption of processed foods and soft drinks containing these additives^2^, nutrient composition tables do not usually include phosphorus from food additives^3^. Therefore, dietary phosphorus intake may have been underestimated^1^.

Many researchers have reported the adverse effects of excess phosphorus intake. High phosphorus (HP) intake reduces bone mineral density (BMD) in rats^4–6^ and adversely affects bone metabolism in humans^7^. HP intake induces arterial medial calcification in animal models of chronic kidney disease (CKD)^8,9^ and impairs endothelial function in humans^10^. An HP diet increases renal calcium concentration and induces high incidence of nephrocalcinosis in rats^11,12^. In a prospective cohort of US healthy adults, HP intake is associated with increased mortality^13^.

Fibroblast growth factor-23 (FGF23) is an important regulator of phosphorus homeostasis by inhibiting renal phosphorus reabsorption and decreasing intestinal phosphorus absorption through inhibiting 1,25-dihydroxyvitamin D [1,25(OH)_2_D] synthesis. Some factors affect FGF23, and one of which is estrogen. An *in vitro* study reported that estrogen increased mRNA expression and protein levels of FGF23 in osteoblast-like cells^14^. Ovariectomy (OVX) decreases circulating FGF23 levels^15^. Estrogen treatment increases circulating levels and mRNA expression of FGF23 in a rat model of CKD with OVX^14^. Therefore, we hypothesized that estrogen may contribute to prevention of the adverse effects of excess phosphorus intake by stimulating FGF23, and the effects of excess phosphorus intake may depend on estrogen status.

This study aimed to investigate the involvement of estrogen in the effects of HP intake on bone metabolism and ectopic calcification and to clarify the interaction between estrogen status and HP intake, and the mechanism. The present study indicated that the effect of HP intake on bone metabolism and aortic calcification did not depend on the estrogen status; in contrast, HP intake synergistically induced nephrocalcinosis in the presence of estrogenic action on the bone, and FGF23 was involved in nephrocalcinosis induced by HP intake partially through FGFR1 signaling.

## Results

### Effects of HP diet on bone metabolism and ectopic calcification in OVX rats (Experiment 1)

To determine the involvement of estrogen status in the effects of HP intake on bone metabolism and ectopic calcification, BMD, aortic calcification, and renal calcification were assessed in sham and OVX rats fed with either a normal phosphorus diet (NP: 0.3% phosphorus, 0.5% calcium) or HP diet (1.2% phosphorus, 0.3% calcium) for 12 weeks. Female rats show regular estrous cycle of 4 or 5 days and plasma estrogen levels vary during the estrous cycle^16^. In contrast, plasma estrogen levels remain higher in sham female rats even in the estrous stage (when plasma estrogen levels are lowest) than in ovariectomized rats^17^. Therefore, in the present study we did not consider the estrous cycle.

**Figure 1:**
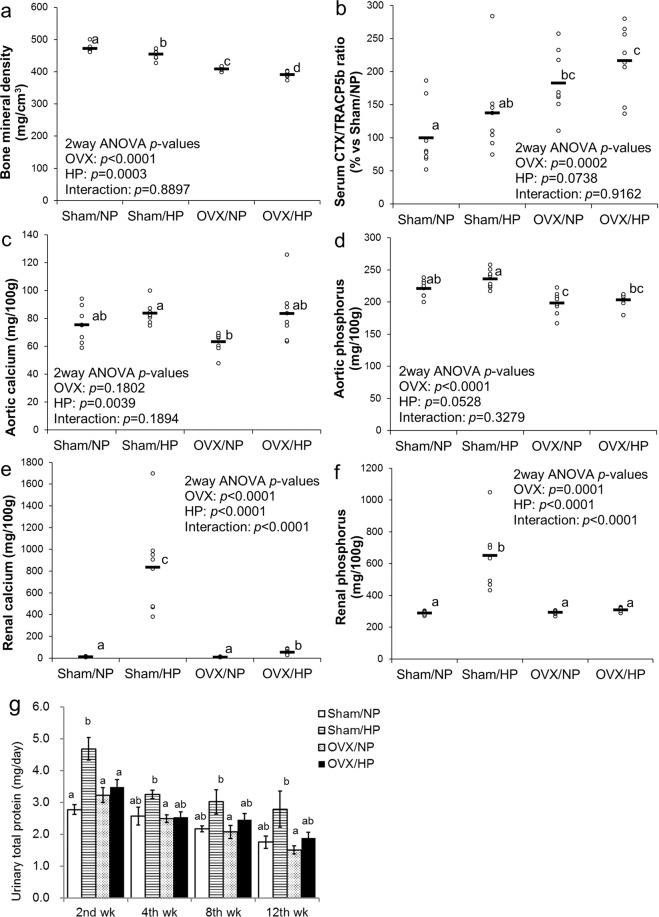
Effects of HP diet on bone metabolism, aortic calcification, and renal calcification in OVX rats (Experiment 1): Scatter plots represent individual rats (n = 8), and horizontal bars through the plots indicate the mean values (**a**–**f**). Columns with error bars represent the mean ± SEM of eight rats. (**g**) Bars or columns with different letters are significantly different (*p* < 0.05). (**a**) Lumbar vertebral bone mineral density. (**b**) Serum CTX/TRACP5b ratio (bone resorption index). (**c**) Abdominal aortic calcium concentration. (**d**) Abdominal aortic phosphorus concentration. (**e**) Renal calcium concentration. (**f**) Renal phosphorus concentration. (**g**) Urinary total protein excretion at 2nd, 4th, 8th, and 12th weeks. NP, normal phosphorus diet; HP, high phosphorus diet; OVX, ovariectomy.

At the end of the experimental period, lumbar vertebral BMD was significantly lower in the OVX treatment group than that in the sham group and was significantly lower in the HP diet group than that in the NP group (Fig. 1a). Rissanen *et al*.^18^ reported that C-terminal cross-linked telopeptides of type I collagen (CTX)/tartrate-resistant acid phosphatase 5b (TRAP5b) is a useful index of bone resorption in the rat OVX model. Serum CTX/TRACP5b ratio was significantly higher in the OVX treatment group than that in the sham group and tended to be higher in the HP diet group than that in the NP group (Fig. 1b). The interaction was not significant in both BMD and bone resorption index, suggesting that HP intake and OVX additively decreased BMD by stimulating bone resorption activity, and the adverse effects of HP intake were independent of estrogen status.

Abdominal aortic calcium concentration in the HP diet group was significantly but slightly higher than that in the NP group (Fig. 1c). OVX treatment and interaction were not significant in terms of aortic calcium. Abdominal aortic phosphorus concentration tended to be higher in the HP diet group than that in the NP group, and significantly lower in the OVX treatment group than that in the sham group (Fig. 1d). The interaction was not significant in terms of aortic phosphorus.

Renal calcium concentration in the sham/HP group was markedly higher than that in the sham/NP group; however, it was modestly higher in the OVX/HP group than that in the OVX/NP group (Fig. 1e). A significant interaction was observed in renal calcium. A similar trend was observed in renal phosphorus concentration (Fig. 1f) and urinary total protein excretion (Fig. 1g), suggesting that HP intake induced nephrocalcinosis, leading to renal damage in the presence of estrogen; however, the absence of estrogen alleviated these adverse effects.

**Figure 2:**
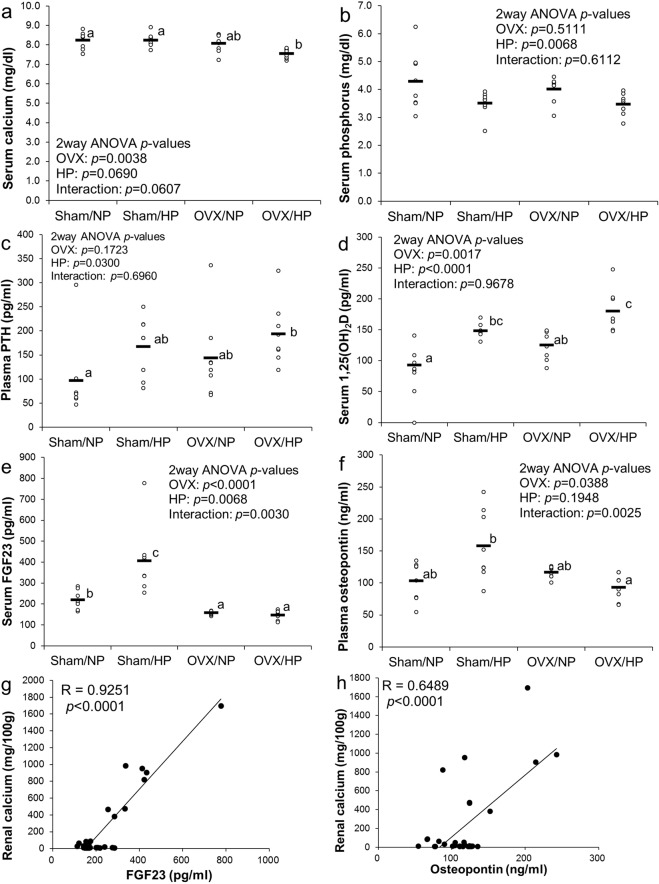
Effects of HP diet on biochemical factors in OVX rats (Experiment 1): Scatter plots represent individual rats (n = 8), and horizontal bars through the plots indicate the mean values (**a**–**f**). Bars with different letters are significantly different (*p* < 0.05). (**a**) Serum calcium levels. (**b**) Serum phosphorus levels. (**c**) PTH levels. (**d**) Serum 1,25(OH)_2_D levels. (**e**) Serum FGF23 levels. (**f**) Plasma osteocalcin levels. Correlations between renal calcium concentration and serum FGF23 levels (**g**) or plasma osteocalcin levels (**h**). NP, normal phosphorus diet; HP, high phosphorus diet; OVX, ovariectomy; 1,25(OH)_2_D, 1,25-dihydroxyvitamin D; FGF23, fibroblast growth factor 23.

Serum calcium levels in the OVX/HP group were significantly lower than those in the sham groups (Fig. 2a). Serum phosphorus levels in the HP group were significantly lower than those in the NP group; however, OVX treatment and interaction were not significant (Fig. 2b). Plasma parathyroid hormone (PTH) levels in the HP group were significantly higher than those in the NP group; however, OVX treatment and interaction were not significant (Fig. 2c). Serum 1,25(OH)_2_D levels in the HP group were significantly higher than those in the NP group and were significantly higher in the OVX group than those in the sham group (Fig. 2d). No significant interaction was observed in both PTH and 1,25(OH)_2_D levels. Interestingly, serum FGF23 concentration in the sham/HP group was significantly higher than that in the sham/NP group; however, it did not differ between the OVX/NP and OVX/HP groups (Fig. 2e). A similar trend was observed in plasma osteopontin levels (Fig. 2f). Furthermore, a linear regression analysis showed a positive correlation between renal calcium concentration and serum FGF23 levels (R = 0.9251, *p* < 0.0001) (Fig. 2g) or plasma osteopontin levels (R = 0.6489, *p* < 0.0001) (Fig. 2h). These findings suggest the involvement of FGF23 and osteopontin in HP-induced nephrocalcinosis.

### Effects of selective estrogen-receptor modulator administration on nephrocalcinosis in OVX rats fed with HP diet (Experiment 2)

Subsequently, we hypothesized that estrogenic action on the bone may influence nephrocalcinosis induced by HP intake by stimulating FGF23, because an *in vitro* study has reported that estrogen increased mRNA expression and protein levels of FGF23 in osteoblast-like cells^14^. Therefore, to further examine the involvement of estrogenic action on the bone in nephrocalcinosis induced by HP intake, OVX rats fed with HP diet were treated with raloxifene, a selective estrogen-receptor modulator (SERM). SERM exerts an estrogenic action on the bone while exhibiting an antiestrogenic action in the breast and uterus^19^. Among SERMs, raloxifene has been widely used in prevention and treatment of postmenopausal osteoporosis. Furthermore, estrogen acts on bone not only in an estrogen receptor-dependent manner but in an estrogen receptor-independent manner, and raloxifene also has the same actions as estrogen^20^. Therefore, we chose raloxifene.

**Figure 3:**
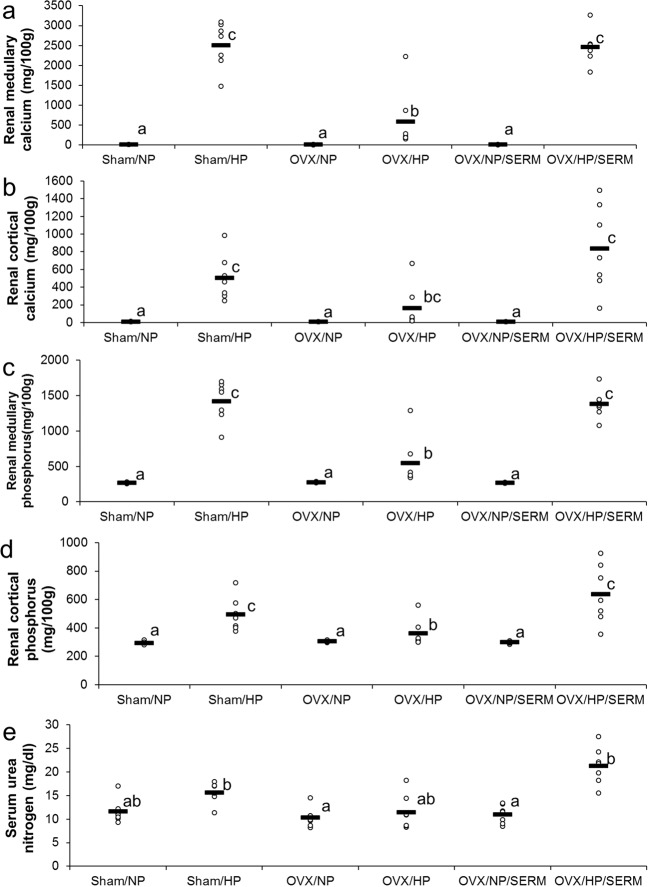
Effects of SERM administration on renal calcification in OVX rats fed with HP diet (Experiment 2): Scatter plots represent individual rats (n = 7), and horizontal bars through the plots indicate the mean values. Bars with different letters are significantly different (*p* < 0.05). (**a**) Renal medullary calcium concentration. (**b**) Renal cortical calcium concentration. (**c**) Renal medullary phosphorus concentration. (**d**) Renal cortical phosphorus concentration. (**e**) Serum urea nitrogen levels. NP, normal phosphorus diet; HP, high phosphorus diet; OVX, ovariectomy; SERM, selective estrogen-receptor modulator.

Renal medullary calcium concentration in the HP groups was significantly higher than that in the NP groups (Fig. 3a) and was significantly higher in the OVX/HP/SERM group than that in OVX/HP group to an extent similar to that in the sham/HP group. A similar trend was observed in renal cortical calcium (Fig. 3b), renal medullary phosphorus (Fig. 3c), renal cortical phosphorus (Fig. 3d), and serum urea nitrogen (Fig. 3e), suggesting that estrogenic action on the bone aggravated nephrocalcinosis induced by HP intake.

**Figure 4:**
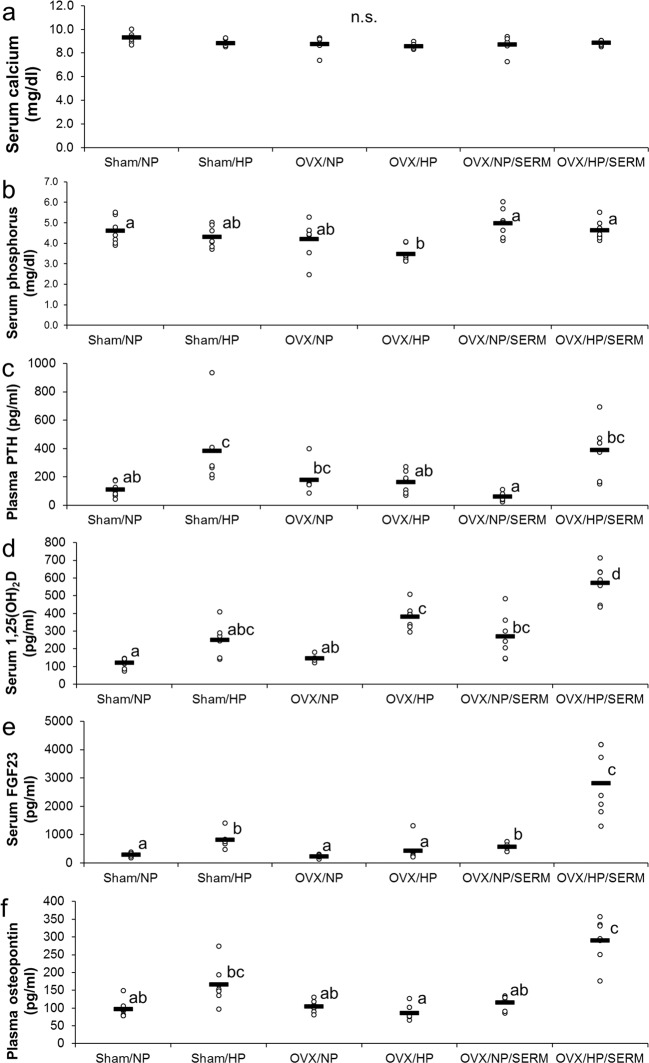
Effects of SERM administration in ovariectomized rats fed with HP diet on biochemical factors (Experiment 2): Scatter plots represent individual rats (n = 7), and horizontal bars through the plots indicate the mean values. Bars with different letters are significantly different (*p* < 0.05). (**a**) Serum calcium levels. (**b**) Serum phosphorus levels. (**c**) Plasma PTH levels. (**d**) Serum 1,25(OH)_2_D levels. (**e**) Serum FGF23 levels. (**f**) Plasma osteocalcin levels. NP, normal phosphorus diet; HP, high phosphorus diet; OVX, ovariectomy; SERM, selective estrogen-receptor modulator; 1,25(OH)_2_D, 1,25-dihydroxyvitamin D; FGF23, fibroblast growth factor 23.

No significant difference was found among the groups in terms of serum calcium levels (Fig. 4a). Serum phosphorus levels in the OVX/HP group were significantly lower than those in the sham/NP, OVX/NP/SERM, and OVX/HP/SERM groups (Fig. 4b). Plasma PTH levels in the HP group were significantly higher than those in the sham/NP, OVX/HP, and OVX/NP/SERM groups, and were significantly higher in the OVX/HP/SERM group than those in the OVX/NP/SERM group (Fig. 4c). HP diet tended to increase or significantly increase serum 1,25(OH)_2_D levels in the sham (*p* = 0.0773), OVX (*p* < 0.05), and OVX/SERM groups (*p* < 0.05) (Fig. 4d). HP diet significantly increased serum FGF23 levels in the sham and OVX/SERM groups (Fig. 4e). Similar findings were observed in osteopontin levels (Fig. 4f), which support the suggestion of Experiment 1 that FGF23 and osteopontin are involved in HP-induced nephrocalcinosis.

### Effects of FGFR1 inhibitor on nephrocalcinosis induced by HP intake (Experiment 3)

**Figure 5:**
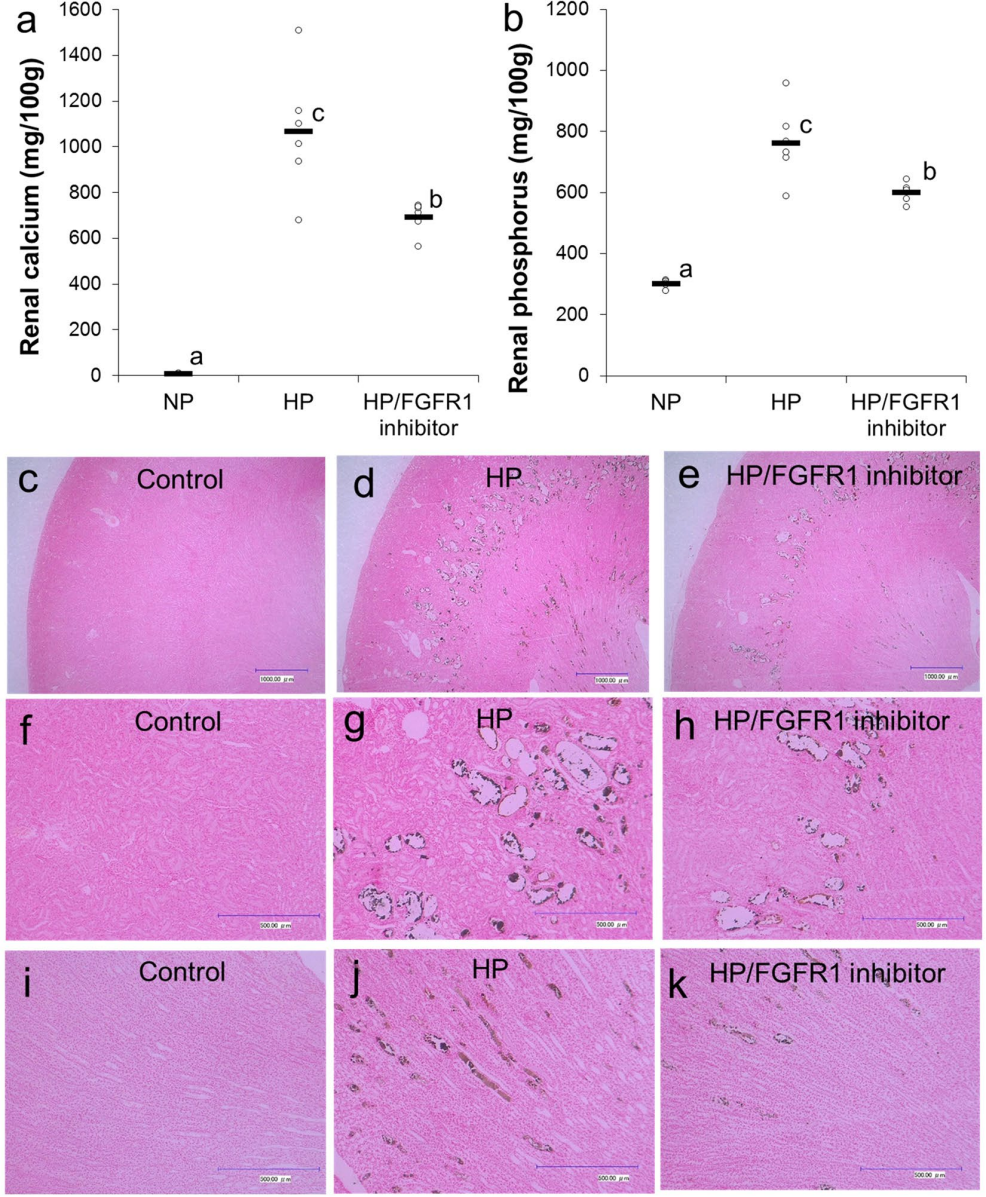
Effects of FGFR1 inhibitor on nephrocalcinosis induced by HP diet (Experiment 3): Scatter plots represent individual rats (n = 6), and horizontal bars through the plots indicate the mean values (**a**,**b**). Bars with different letters are significantly different (*p* < 0.05). (**a**) Renal calcium concentration. (**b**) Renal phosphorus concentration. Representative images of Von Kossa staining of longitudinal section of the kidney tissues in the NP group (c, f, i), HP group (**d**,**g**,**j**) and HP/FGFR1 inhibitor group (**e**,**h**,**k**). Original magnification, ×50 (**c**–**e**) or ×200 (**f**–**k**). Bar = 1000 µm (**c–e**) or 500 µm (**f**–**k**). NP, normal phosphorus diet; HP, high phosphorus diet; FGFR1, fibroblast growth factor receptor 1.

Fibroblast growth factor family members bind to four known FGFRs and initiate intracellular signaling^21^. Some researchers reported that FGFR1 was the predominant receptor of FGF23^22,23^. Therefore, we investigated the involvement of FGFR1 signaling in nephrocalcinosis induced by HP diet in female rats. Renal calcium concentration in the HP group was significantly higher than that in the NP group and was significantly lower in the HP/FGFR1 inhibitor group than that in the HP group (Fig. 5a). The concentration in the HP/FGFR1 inhibitor group showed intermediate levels between the NP and HP groups. A similar trend was observed in renal phosphorus concentration (Fig. 5b). Representative images of Von Kossa staining of the longitudinal section of the kidney tissues are shown in Fig. 5c–k. The images revealed that calcium deposits were located in the renal tubule in the HP group, and FGFR1 inhibitor treatment partially recovered the effects of HP, particularly in the medulla. These results suggest that FGF23 was involved in nephrocalcinosis induced by HP intake partially through FGFR1 signaling.

## Discussion

This study investigated the involvement of estrogen in the effects of HP intake on bone metabolism and ectopic calcification. The present study indicated that HP intake additively decreased BMD in the absence of estrogen through stimulating bone resorption activity. Koshihara *et al*.^5^ reported that HP intake reduced BMD by increasing bone turnover in OVX rats, which is consistent with our results.

HP intake slightly increased aortic calcium concentration irrespective of OVX treatment. Park *et al*.^24^ reported that OVX alone did not induce aortic calcification, which supports our results. Our findings suggest that the effects of HP intake on aortic calcification are not dependent on the estrogen status. A previous study reported that an HP diet induced arterial medial calcification and elevated FGF23 levels in animal models of CKD, and arterial calcification was strongly correlated with serum FGF23 levels even when serum phosphorus levels are normal, suggesting the possibility that FGF23 might play roles as a marker and/or mediator of vascular calcification^8^. In contrast, the present study did not find any correlation between arterial calcium concentration and FGF23 levels.

Quantitative and histopathological analyses revealed that HP intake induced intraluminal calcium phosphate deposits in renal tubules (nephrocalcinosis). These findings are consistent with previous reports^11,12^. HP intake synergistically induced nephrocalcinosis in the presence of estrogen. Furthermore, the present study showed that nephrocalcinosis induced by HP intake was alleviated by OVX treatment, and the effect was reversed by SERM (raloxifene) administration. Raloxifene binds to the estrogen-receptor with almost the same affinity as estrogen and exerts an estrogenic action on the bone while exhibiting an antiestrogenic action in the breast and uterus^19^. These suggest that nephrocalcinosis induced by HP intake is dependent on estrogenic action on the bone.

**Figure 6:**
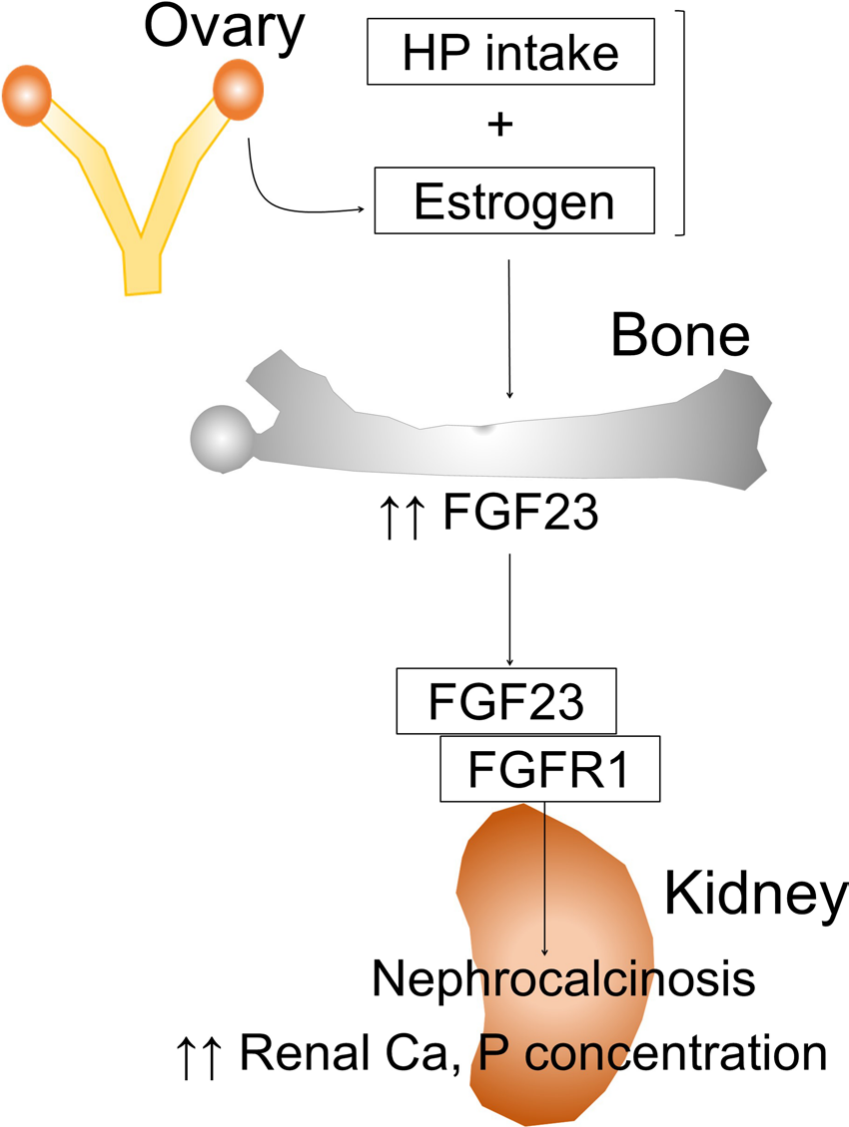
Schematic diagram of the hypothesized mechanism by which HP intake in the presence of estrogen induces nephrocalcinosis: HP, high phosphorus; FGF23, fibroblast growth factor 23; FGFR1, fibroblast growth factor receptor 1; Ca, calcium.

Interestingly, the present study also indicated a strong positive correlation between renal calcium concentration and FGF23 or osteopontin levels. Paloian *et al*.^25^ reported that osteopontin plays a critical role in the prevention of phosphorus-induced nephrocalcinosis. Therefore, osteopontin may be elevated as a compensatory effect to prevent nephrocalcinosis induced by HP intake. In the present study, FGFR1 inhibitor treatment partially recovered nephrocalcinosis induced by HP intake. Fibroblast growth factor family members bind to four known FGFRs and initiate intracellular signaling^21^. FGFR1 was reported to be the predominant receptor of FGF23^22,23^. Our findings suggest that FGF23 is involved in nephrocalcinosis induced by HP intake partially through FGFR1 signaling. A previous *in vitro* study reported that estrogen increased mRNA expression and protein levels of FGF23 in osteoblast-like cells^14^. Therefore, estrogenic action on the bone aggravates nephrocalcinosis induced by HP intake, possibly through stimulating FGF23 in the bone (Fig. 6). The mechanism by which FGF23/FGFR1 signaling aggravates nephrocalcinosis induced by HP intake remains to be clarified. Further studies are necessary to clarify the mechanism.

There are several types of genetic and acquired FGF23-related hypophosphatemic diseases. Among these diseases, X-linked hypophosphatemia (XLH), which is caused by inactivating mutations in the phosphate-regulating endopeptidase homolog, X-linked gene, is the most prevalent form of genetic FGF23-related hypophosphatemic rickets^26^. Patients with XLH show local and systemic symptoms, including hypophosphatemia, elevated FGF23 levels, impaired growth, rickets, osteomalacia, bone abnormalities, bone pain, spontaneous dental abscesses, hearing difficulties, enthesopathy, osteoarthritis, and muscular dysfunction^27^. In patients with XLH, oral phosphate and calcitriol or calcitriol analogs are used as conventional therapy. However, conventional therapy has been associated with adverse effects, including nephrocalcinosis^28^. Our results may partly explain the mechanism by which oral phosphate therapy induces nephrocalcinosis in patients with XLH. In brief, the simultaneous presence of HP loading and high FGF23 levels may be a risk factor for nephrocalcinosis in patients with XLH. In fact, nephrocalcinosis has not been reported in anti-FGF23 antibody therapy in patients with XLH^29^, which supports our findings.

Phosphorus is one of the nutrients that are more likely to be consumed excessively as a result of the increasing use of phosphorus-containing food additives and increasing consumption of processed foods and soft drinks containing these additives^2^. Furthermore, oral sodium phosphate treatment is commonly used to cleanse the bowel during preparation for colonoscopy^30–32^. Therefore, HP loading is a common problem. Some researchers reported that phosphate-induced nephrocalcinosis is an under-recognized cause of acute kidney injury that potentially leads to CKD^33–35^. In the present study, rats fed with HP diet had severe nephrocalcinosis even when serum calcium and phosphorus levels were in the normal or lower range, suggesting that serum calcium and/or phosphorus levels cannot be a biomarker of phosphate-induced nephrocalcinosis. In contrast, we found a strong positive correlation between renal calcium concentration and FGF23 levels. Isakova *et al*.^36^ reported that serum FGF23 might be a sensitive early biomarker of disordered phosphorus metabolism in patients with CKD and normal serum phosphate levels. Serum FGF23 might also serve as a biomarker of phosphate-induced nephrocalcinosis.

In conclusion, the present study showed that the effect of HP intake on bone metabolism and aortic calcification did not depend on estrogen status; in contrast, HP intake synergistically induced nephrocalcinosis in the presence of estrogenic action on the bone, and FGF23 was involved in nephrocalcinosis induced by HP intake partially through FGFR1 signaling.

## Methods

### Animals and diets

In Experiment 1, 32 10-week-old female Wistar rats were purchased from SLC Japan (Shizuoka, Japan). After 2-week adaptation, the rats were divided into two groups: the sham-operated and OVX groups. Each group was divided into two subgroups postoperatively: a group fed with a control diet containing NP content (0.3% phosphorus, 0.5% calcium) and a group fed with HP diet (1.2% phosphorus, 0.5% calcium) (n = 8 for each subgroup). In Experiment 2, 42 11-week-old female Wistar rats were purchased from SLC Japan. After a week of adaptation, the rats were divided into two groups: the sham and OVX groups. The sham group was divided into two subgroups postoperatively: those fed with NP and HP diet. The OVX group was divided into four subgroups: those fed with NP diet, HP diet, NP diet with 3 mg/kg BW raloxifene HCl (Evista: Eli Lilly, Kobe, Japan), and HP diet with 3 mg/kgBW raloxifene HCl (n = 7 for each subgroup). In Experiment 3, 18 12-week-old female Wistar rats (SLC, Japan) were divided into three groups: those fed with NP diet (NP group), HP diet (HP group), and HP diet with 1 mg/kgBW FGFR1 inhibitor (HP/FGFR1 inhibitor group) (n = 6, for each group). The HP/FGFR1 inhibitor group was intraperitoneally injected with 1 mg/kgBW FGFR1 inhibitor (PD173074: Sigma-Aldrich, Japan) once daily, and the control and HP groups were injected with an equivalent volume of vehicle (saline solution containing 0.5% DMSO) in the same manner. All the groups were allowed free access to their respective experimental diets and water for 12 weeks (Experiments 1 and 2) and 4 weeks (Experiment 3). All rats were individually housed in stainless steel metabolic cages in a temperature, humidity, and light-controlled room (21 °C ± 2 °C, 55% ± 15% humidity, 12-h light/dark cycle) and cared for in accordance with the guidelines of the ethics committee on animal use of Meiji Corporation Ltd. and relevant Laws (no. 105, 1973) and Notifications (no. 6, 1980) of the Japanese Government (Ethics approval code: No. 2011_3871_0105, No.2012_3871_0087, No.2013_3871_0107).

### BMD by X-ray computed tomography analysis

In Experiment 1, BMD was measured using an X-ray computed tomography system (LaTheta LCT-100 M; Hitachi Aloka Medical, Ltd., Tokyo, Japan). Contiguous 1.0-mm slices for the 2nd to 4th lumbar vertebrae (L2–L4) were utilized for quantitative measurement. Total BMD was analyzed using LaTheta software (Version 1.31).

### Sample collections

At the 2nd, 4th, 8th, and 12th weeks of measurement of total protein excretion, 24-h urine samples of each rat were collected (Experiment 1). In Experiments 1, 2, and 3, blood samples were obtained from the abdominal aorta under anesthesia at the end of the experimental period. An aliquot of the blood was immediately transferred to tubes containing ethylenediaminetetraacetic acid, and the other was transferred to serum tubes. The serum and plasma samples were separated by centrifugation at 3000 *g* for 15 min at 4 °C and stored at −80 °C until analysis. After the rats were killed, the right kidney (Experiments 1, 2, and 3), the left kidney (Experiment 3), and abdominal aorta (Experiment 1) were excised. In Experiment 2, the kidney was divided into two parts: the medulla and cortex. The right kidney, renal medulla, renal cortex, and abdominal aorta were preserved at −80 °C until the analysis of calcium and phosphorus concentrations. The left kidney was fixed in 10% neutral-buffered formalin (pH 7.4; Wako Pure Chemical Industries Co., Osaka, Japan).

### Serum, plasma, and urine biochemistry

Serum FGF23 levels were determined using the FGF23 enzyme-linked immunosorbent assay (ELISA) kit (Kainos Laboratories, Tokyo, Japan). Serum calcium, phosphorus, and urea nitrogen levels and urinary protein excretion were measured using commercial kits (Wako Pure Chemical Industries, Co.). Plasma intact PTH levels were assayed using the Rat Bioactive Intact PTH ELISA kit (Immunotopics International, San Clemente, CA, USA). Plasma osteopontin levels were assayed using the Rat Osteopontin Assay Kit (IBL, Gunma, Japan). Serum CTX and TRACP5b were measured using the RatLaps EIA kit and RatTRAP Assay kit, respectively, both of which were manufactured by Immunodiagnostic Systems Nordic A/S (Herlev, Denmark). The resorption index was calculated as the CTX/TRACP5b ratio based on the previous report^18^. Serum 1,25(OH)_2_D levels were determined using radioimmunoassay (Mitsubishi Chemical Medience Corporation, Tokyo, Japan).

### Quantitative biochemical analysis of aortic and renal calcium and phosphorus

The samples of the right kidney, renal medulla, renal cortex, and abdominal aorta were mineralized in trace element-grade concentrated nitric acid (Wako Pure Chemical Industries, Co.) by using a microwave system (Multiwave3000; Perkin Elmer, Tokyo, Japan) and analyzed for calcium and phosphorus by using inductively coupled plasma spectroscopy (ICPE-9000; Shimadzu, Kyoto, Japan).

### Histopathology

The samples of formalin-fixed kidneys were embedded in paraffin. The kidneys were longitudinally sliced and stained with Von Kossa based on standard methods. Imaging of stained sections was performed using a digital microscope (VHX-600: KEYENCE, Osaka, Japan).

### Statistics

Data other than urinary total protein excretion are expressed as scatter plots and horizontal bars. Scatter plots represent individual rats, and horizontal bars through the plots indicate the mean values. Data in urinary total excretion are expressed as mean ± standard error of the mean. In Experiment 1, treatment effects were analyzed using two-way analysis of variance (OVX × HP). In Experiments 1, 2, and 3, Tukey–Kramer test or Steel–Dwass multiple comparison test was used to detect significant differences among the groups. A correlation coefficient between renal calcium and serum biomarkers was calculated using the least-squares method using Microsoft Excel 2016 (Microsoft, Tokyo, Japan). Moreover, *p* < 0.05 was considered significant. All statistical analyses were performed using BellCurve for Excel software (Social Survey Research Information Co., Ltd., Tokyo, Japan).

## Data Availability

The data sets generated during the current study are available on reasonable request.
